# Efficacy of Topical Analgesics in Pain Control for Corneal Abrasions: A Systematic Review

**DOI:** 10.7759/cureus.1121

**Published:** 2017-03-27

**Authors:** Bryan Thiel, Alexandra Sarau, Daniel Ng

**Affiliations:** 1 University of Central Florida, College of Medicine

**Keywords:** corneal abrasions, anesthetics, cycloplegics, nsaids, pain management

## Abstract

Corneal abrasions are one of the most common ocular injuries seen in the emergency department. While most patients with corneal abrasions complain of excruciating pain, permanent sequelae may develop if not managed properly. The use of topical antibiotics and other standards of treatment have greatly reduced the incidence of complications. However, there is still a lack of consensus regarding the proper management of pain in corneal abrasions. Proposed analgesics for the control of corneal abrasion pain include topical nonsteroidal anti-inflammatory drugs (NSAIDs), topical anesthetics, and topical cycloplegics. For this review, ten published randomized controlled trials were identified, focusing on the efficacy and safety of different topical analgesics used in treating corneal abrasions.  Six of the trials focused on topical NSAIDs, three on topical anesthetics, and one on topical cycloplegics. There were mixed results regarding the efficacy of topical analgesics in reducing pain in patients with corneal abrasions. This review of the literature revealed that topical NSAIDs produced reductions in pain symptoms, whereas topical anesthetics and cycloplegics did not demonstrate significant improvements in either healing rates or pain control. Thus, this evidence supports the use of topical NSAIDs in the standard management of corneal abrasions. Unfortunately, the power of these studies is largely limited by small sample sizes. Larger studies must be conducted before topical analgesics can be recommended or discouraged for pain management in corneal abrasions. However, based on this review of the literature, the use of topical NSAIDs does not appear to complicate wound healing, and thus remains a safe option in patients desiring medical treatment.

## Introduction and background

Corneal abrasions account for a large portion of eye-related injuries seen with an incidence of approximately 3 in 1000 persons presenting to the emergency room [1]. Although these ocular injuries occur in all age groups, young, working males typically have the highest rates of occupational eye-related injuries. Corneal abrasions result from trauma to the corneal epithelium and present with varying symptoms including severe acute pain, excessive lacrimation, foreign body sensation, photophobia, blepharospasm, and blurred vision in the affected eye. If not diagnosed and treated properly, potential complications such as superinfection, corneal perforation, scarring, or infectious keratitis may result [2].  Because the ocular pain can be debilitating and disrupt daily functioning, pain control is an important aspect of management in these patients.

In the past, corneal abrasions were traditionally treated with eye patching [3]. It was believed that this method would reduce blinking and corneal trauma secondary to eyelid closure, thereby reducing pain. However, eye patching is no longer recommended due to lack of evidence of improvement in either pain or healing rate [4, 5]. In fact, evidence shows that applying an eye patch to corneal abrasions decreases oxygen delivery and results in higher risks of infection due to increased moisture [4]. Current treatment options for corneal abrasions include topical analgesics, topical anesthetics, topical cycloplegics (also known as mydriatics), and topical antibiotics. However, the efficacies and safety profiles of these therapeutic options remain unclear, and the protocol for topical intervention is still controversial. Topical nonsteroidal anti-inflammatory drugs (NSAIDs) have been shown to significantly reduce pain scores at 24 hours after the start of intervention when compared with placebo drops [2]. In addition, studies have demonstrated a significant reduction in supplementary oral analgesic use when ophthalmic NSAIDs were applied [5, 6]. Unfortunately, studies on the benefits of ophthalmic interventions on pain reduction and recovery time are lacking, and the existing literature is limited by small sample sizes [5, 7].  A study by Waldman et al. demonstrated higher patient-reported effectiveness of topical tetracaine, a local anesthetic, compared to saline; the research group recommended that short-term local anesthetic use become a standard practice of corneal abrasion management [8]. In contrast, a meta-analysis by Puls et al. found no differences in pain rating, symptoms, or healing rate when comparing topical anesthetic and placebo treatment, thus discouraging outpatient use [9]. Furthermore, adverse events have been associated with topical intervention when managing patients with corneal abrasions. Long-term topical ophthalmic NSAID use has been shown to result in negative outcomes, including contact dermatitis, conjunctival hyperemia, transient stinging, and corneal ulcerations [2, 10]. Meek et al. reported that cycloplegics have the potential to precipitate acute glaucoma as well as other systemic anticholinergic symptoms [7]. Ultimately,  the benefit-to-risk ratio of topical ophthalmic interventions remains unclear. The purpose of this literature review is to perform an analysis of current available research and to determine the effectiveness of topical analgesics on reducing pain in patients with corneal abrasions.

## Review

### Nonsteroidal Anti-Inflammatory Drugs (NSAIDs)

This review seeks to determine which topical analgesic is best supported by literature to provide pain relief following patients with corneal abrasions. We have separated the review by drug type starting with NSAIDs as demonstrated in Table 1.

**Table 1:** Study Characteristics and Outcomes of NSAID Trials

Paper Title	Author	Design	Number of Patients	Number on Intervention	Number on Control	Intervention	Outcomes	Follow-up	Results
Randomised controlled trial of ketorolac in the management of corneal abrasions.	Goyal et al. [5]	Randomized controlled trial	85	43	42	Ketorolac trometamol 0.5% ophthalmic solution used 4 times a day	Pain, photophobia, grittiness, watering, blurring of vision, need for additional analgesics, duration of pain, effect on sleep	Questionnaire given before treatment and repeated 1 day after	Number of patients requiring additional oral analgesics less in intervention (7 vs. 21) (P = .002)
The Effectiveness of Topical Diclofenac in Relieving Discomfort Following Traumatic Corneal Abrasions	Jayamanne et al. [11]	Randomized controlled trial	40	20	20	Diclofenac sodium 0.1% ophthalmic solution	Categorical pain scale ranging from none to severe disabling pain, foreign body sensation, light sensitivity, headache-like pain	Questionnaire given on day 0, 1 and 2	Significant reduction in pain in the intervention group (P < .002); increased frequency in foreign body sensation, light sensitivity and headache-like pain in the control group
Safety and Efficacy of Diclofenac Ophthalmic Solution in the Treatment of Corneal Abrasions	Szucs et al. [6]	Randomized controlled trial	49	25	24	Diclofenac sodium 0.1% ophthalmic solution	Pain measured using visual Numeric Pain Intensity Scale (NPIS) and need for rescue medication to manage analgesia	NPIS score 1-10 two hours after treatment via phone call	Greater reduction in pain in the intervention group after 2 hours, (95% CI, 0.8-3.4)
Combined indomethacin/gentamicin eyedrops to reduce pain after traumatic corneal abrasion.	Alberti et al. [12]	Randomized controlled trial	123	62	61	Combined indomethacin 0.1%/gentamicin sulphate 300,000 IU/100 mL eyedrops given 4 times daily	Pain measured using visual analog scale (VAS), photophobia, tearing, burning, irritation, foreign body sensation, conjunctival hyperemia, ciliary injection, adverse effects, tolerance	Evaluations before treatment, 1 hour after first treatment instillation, 1 hour after second treatment instillation, day 1 after treatment, and day 4/5 after treatment	Treatment effect 1 hour after first treatment instillation greater in interven tion than control (*P* = .007); improved time course of pain in treatment group (P = .015)
Evaluation of the Analgesic Effect of 0.1% Indomethacin Solution on Corneal Abrasions	Patrone et al. [13]	Randomized controlled trial	347	178	169	Treated with 0.3% netilmicin and 0.1% indomethacin eye drops	Pain measured via verbal pain scale	Verbal pain scale evaluated 30 min after receiving treatment, after 12 h, and after 24 h	Greater reduction of subjective pain in intervention group after 12 h (P < .0001) and 24 h (P < .0001)
Topical analgesia for superficial corneal injuries	Brahma et al. [3]	Randomized controlled trial	244	109 (2 intervention groups)	115 (2 control groups)	Treated with homatropine 2% drops and flurbiprofen 0.03% drops four times daily for 48 hours	Pain measured using VASrating from 0-10. Dichotomous yes/no questions regarding whether additional oral analgesia was used, sleep disturbed, whether patients took time off from work	Questionnaires provided at initial treatment and 6, 12, 18, 24 hours after treatment	Flurbiprofen treated groups had reduced subjective pain scores (*P *< 0.05) during the initial treatment and during each subsequent time period. There was no difference in pain scores between the two treatment groups (*P *> .05). Flurbiprofen receiving groups reported less adjunctive oral analgesia compared to control groups (*P *< .01) and had better sleep (*P *< .05).

The study published in 2001 by Goyal et al. sought to determine the efficacy of the topical NSAID ketorolac trometamol 0.5% solution on pain reduction and healing following corneal abrasion [5]. This study assessed 88 patients based on whether they fit the eligibility criteria which required the following: 1) either a corneal abrasion or foreign body removal within the past 48 hours, 2) age between 16-80, and 3) no prior treatment. Participants were excluded from the study if they wore contact lenses, had signs of an infection, had an erosion affecting greater than one-third the surface of the cornea, or if they had a past history of corneal disease (i.e. corneal dystrophy). Patients were randomly assigned to receive either the ketorolac solution or the control of Liquifilm tears after which they were assessed for subjective symptoms which included pain. When assessed for pain, there was no significant difference between the groups (P = .28) but patients in the treatment group required statistically significantly less supplementary oral analgesics to control pain (P = .002). While subjective pain of the patient was not necessarily affected by the use of topical NSAIDs, indirect measurement through the need for additional oral analgesics demonstrated that ketorolac may provide increased relief in comparison to the Liquifilm tears solution. This study also attempted to assess whether ketorolac could provide relief for other symptoms such as photophobia, grittiness, watering or blurring of the eyes, but there was no difference between treatment and the control groups for these parameters. Furthermore, there was no difference in the rate of epithelial closure between the groups.  Overall, while there was no difference in subjective pain, the treatment group required less oral analgesia to manage the pain.

Diclofenac is another ophthalmic NSAID that has been studied and published with results regarding pain management. In a study of 40 patients with corneal abrasions, participants were asked to provide visual and categorical pain scores over a period of three days [11]. The participants in the treatment group received 0.1% Diclofenac solution to administer topically while the control received normal saline. There was no significant reduction of pain between the treatment group and the control group on the same day of treatment. However, for pain outcomes on day 1 and 2 after treatment, patients who received Diclofenac were less likely to state their pain as “severe” or “moderate,” instead favoring “none” or “mild” (*P *< .02). Szucs et al. assessed the effectiveness of Diclofenac solution for corneal abrasions in the emergency department  by recruiting 49 patients for a double-blinded study [6]. Szucs et al. only assessed for immediate, same-day analgesia efficacy. They assessed pain using a visual Numeric Pain Intensity Scale (NPIS) where patients rated their pain on a scale of 1-10 before and after receiving treatment. Patients that received the Diclofenac solution had decreased their NPIS scale by 3.1 (95% CI, 2.3-4.0) compared to the control that had a decrease of 1.0 (95% CI, 0.1-2.0). The difference between the two groups was 2.1±1.3 (95% CI, 0.8-3.4). Szucs et al. also measured a secondary variable for pain assessment: the need for rescue medication of oxycodone-acetaminophen to maintain analgesia. There were no differences in the utilization of rescue medication between the treatment and control groups. While there have been limited studies on Diclofenac ophthalmic solutions for the use of analgesia in patients with corneal abrasions, these two studies have shown that patients report a decrease in pain with the use of the medication. These studies may be affected by very low sample sizes (*n* = 40 and *n* = 49, respectively), therefore larger sized studies may be required before this drug is recommended above others for the standard of treatment for corneal abrasions.

There are two published studies that have assessed the role of indomethacin in corneal abrasions. Alberti et al. evaluated 123 patients in a double-blind, multicenter, randomized controlled trial that assessed whether indomethacin 0.1% and gentamicin given in combination would lead to decreased pain and associated symptoms in patients with traumatic corneal abrasions [12]. Analysis of variance (ANOVA)-adjusted statistics demonstrated greater improvement in pain after one hour (*P *= .007) with the NSAID solution compared to the control group receiving gentamicin eye drops without indomethacin. However, the significance of pain reduction after days 1-3 is not clear. No p-value is provided for other reported times. Limitations  for this study include the following: major differences between treatment groups including distribution of iris colors (*p *= .026), corneal lesion depths (*P *= .05), conjunctival hyperemia (*P *= .001) and associated symptom pain scores (*P *= .03). These statistical differences between groups may limit the strength of association between the indomethacin treatment and reduced pain scores. Patrone et al. further extrapolated on the role of indomethacin 0.1% in a study that assessed for its analgesic properties in traumatic corneal abrasions. In this randomized, blind-unspecified study, 347 patients with traumatic corneal abrasions were provided either indomethacin 0.1% with netilmicin drops 0.3% or the control consisting of just the antibiotic drops [13]. Patient pain was evaluated using a verbal pain scale (VPS) that was measured at 30 minutes, 12 hours, and 24 hours after receiving medication. The verbal pain score was measured on a scale of 1-10, where each number was  given a qualitative descriptor of the pain. For example, a score of 1 indicated that the pain was non-existent, 5 indicated modest pain where it could  interfere with work that needed accuracy, while a 10 was intolerable pain that produced total incapacity. There was no significant difference in pain reduction between the two groups after the first 30 minutes. However, there was a statistically significant decrease in pain in both groups after the 12 hour and 24 hour marks; the decrease was more pronounced in the treatment group receiving indomethacin 0.1% (*P *≤ .0001). There was no significant difference in delay of corneal healing in either group. This study used linear regression analysis which demonstrated that there is a relationship between the dimension of the abrasion at presentation and the level of pain that was elicited.  At 12 hours after receiving treatment, there was no longer a correlation between  pain and the size of abrasion in the treatment group, whereas in the control group, pain continued to be  worse relative to the size of the abrasion. This relationship suggests that indomethacin provided relief to the patient independent of the size of abrasion.

A 1996 study by Brahma et al. examined the effects of flurbiprofen 0.03%, a topical NSAID, on pain relief in superficial corneal injuries, including corneal foreign bodies and abrasions [3]. A total of 224 patients completed the study and were randomly assigned into one of four treatment groups which included the following: polyvinyl alcohol solution (control, group 1), homatropine 2% (group 2), flurbiprofen 0.03% (group 3), or homatropine 2% followed by flurbiprofen 0.03% (group 4). 115 patients were assigned to the control and normal practice groups (groups 1 and 2, respectively), and 109 patients were assigned to the flurbiprofen treatment group (groups 3 and 4). These interventions were administered for 48 hours, and pain was assessed using a visual analogue scale (VAS). Pain scores were collected every six hours for 24 hours.The results demonstrated a significant reduction in pain scores for the two groups receiving flurbiprofen at every time point. (*P *< .05). Additionally, the patients that received flurbiprofen took less oral analgesia than the controls (*P *< .01), had fewer sleep disturbances (*P *< .05), and took less time off work (*P *< .01). Brahma et al. asked 21 of the patients in the treatment groups to report significant adverse effects; no complications related to the topical NSAID were reported. Flurbiprofen eye drops were found to be beneficial and significantly improve pain relief in patients with superficial corneal injuries.

### Topical Anesthetics

Topical anesthetics are another class of ocular intervention that has been used to provide pain relief for corneal abrasions. These results are demonstrated in Table 2.

**Table 2:** Study Characteristics and Outcomes of Topical Anesthetic Trials

Paper Title	Author	Design	Number of Patients	Number on Intervention	Number on Control	Intervention	Outcomes	Followup	Results
Management of ocular trauma in emergency (MOTE) trial: A pilot randomized double-blinded trial comparing topical amethocaine with saline in the outpatient management of corneal trauma	Ting et al. [14]	Randomized controlled trial	47	22	25	1 drop of 0.4% amethocaine applied once hourly	Healing, use of oral analgesics, pain using 10 cm VAS	Evaluation before treatment, 36-48 hours after recruitment; patient diary; telephone interview after recruitment	No effect of treatment on healing. Use of oral analgesics, or pain when compared to control
Dilute proparacaine for the management of acute corneal injuries in the emergency department	Ball et al. [15]	Randomized controlled trial	33	15	18	2-4 drops of 0.05% proparacaine as needed over 7 days	Pain using 10 cm VAS, satisfaction, wound healing, corneal thickness, ophthalmic pathology	Evaluation before treatment and 5 min after treatment; in-person follow-up 1, 3, 5 days after treatment	Efficacious for pain reduction, no complications or delayed wound healing
Topical tetracaine used for 24 hours is safe and rated highly effective by patients for the treatment of pain caused by corneal abrasions: A double-blind, randomized clinical trial	Waldman et al. [8]	Randomized controlled trial	116	59	57	One drop 1% tetracaine hydrochloride every 30 min during first 24 hours of presentation	Pain using 10 cm VAS, patient-perceived effectiveness on a numeric rating scale of 0-10, healing rate	VAS recorded every 2 hours while awake for first 48 hours after treatment, telephone interview	No difference in pain, but treatment was perceived to be more effective, no impairment of wound healing

In 2009, Ting et al. published an article assessing both the efficacy and safety of topical amethocaine in the management of corneal abrasions [14]. 47 total patients completed the trial held at Mater Adult Hospital Emergency Department. These patients were required to have one of three conditions: (1) traumatic superficial corneal abrasion, (2) superficial corneal abrasion with retained foreign body, or (3) keratitis from welding flash exposure. The exclusion criteria were the following: more than 36 hours had passed since the initial event, age younger than 18, history of adverse effects to topical anesthetics or underlying eye pathology not including refractive error, contact lens usage, pregnant or lactating, presence of conjunctival infection, functionally one-eyed, or required an urgent ophthalmologic referral. Patients were randomly assigned to two groups; the control group received 0.9% saline solution while the treatment group received 0.4% amethocaine solution. Both groups applied 1 drop of solution once hourly as needed for pain relief. The option of adjunctive oral analgesics was offered to both groups. The results were inconclusive in determining the effects of amethocaine on wound healing due to the small sample size. As a result, no p-value could be generated. The data showed that 2 out of 7 patients had a persistent corneal defect in the treatment group compared to 1 in 9 patients in the control group. The additional use of oral analgesics, self-reported visual problems, need for unscheduled medical review, and patient satisfaction were not statistically different between both groups. Lastly, of the patients that completed pain diaries, those in the treatment group reported lower pain burdens (*n* = 12, M = 404±75 mm) compared to the control group (*n* = 9, M = 629±172) on VAS pain rating. Pain ratings were scored from 0 mm (no pain) to 100 mm (worst pain imaginable). These scores were taken every 3 hours for 36 hours, creating a cumulative pain score based on 12 pain assessments for each patient. However, the clinical significance of these findings is hindered by the low power secondary to small sample size.

A study released in 2010 by Ball et al. investigated the efficacy of proparacaine in the management of corneal insult [15]. A total of 33 patients recruited in 2005 completed the study which was performed at two tertiary care emergency departments in London, Ontario. Patients were excluded if they demonstrated any of the following characteristics: inability to consent, allergy to proparacaine, inability to follow-up, or presence of any pre-existing eye pathology. The 33 patients were randomly assigned to one of two groups; 18 patients assigned to the control group received 2-4 placebo drops which were administered as needed over the course of 7 days. The remaining 15 patients assigned to the treatment group applied 2-4 drops of 0.05% proparacaine over the course of 7 days as needed. Both groups received topical gatifloxacin to be used 1-2 drops every 2 hours in addition to 325 mg acetaminophen/30 mg codeine tablets as needed for additional pain relief. Although the sample size was small and predominantly male, statistically significant data were obtained. Using a 10 point VAS, the treatment group demonstrated a median improvement of 3.9 (IQR = 1.5-5.1) while the control group demonstrated a median improvement of 0.6 (IQR = 0.2-2.0) (*P *= .007).  The satisfaction of patients was also evaluated using a 10 point VAS; the treatment group had a median satisfaction of 8.0 (IQR = 6.0-9.0) compared to that of the control group median satisfaction of 2.6 (IQR = 1.0-8.0) (*P* = 0.027). Proparacaine was effective at short-term pain reduction and was perceived by patients to be more efficacious as well.

Another study released in 2014 by Waldman et al. examined the use of tetracaine in the management of corneal abrasions [8]. The study took place over the course of 12 months in an emergency department in New Zealand where 116 patients presenting with uncomplicated corneal abrasions were recruited and completed the trial. These patients were randomized into two groups with 57 assigned to the control group and 59 assigned to the treatment group. Exclusion criteria included the following: presentation after 36 hours of initial injury, age under 18, history of past eye surgeries/cataracts, contact lens use, deafness, inability to consent, injury to both eyes, co-existing ocular conditions or infections, allergy to tetracaine or similar medications, urgent presentation, or inability to follow-up.  In this double-blinded, randomized trial,  the control group received saline and applied one drop every 30 minutes during the first 24 hours after presentation to the ED. The treatment group was given 1% tetracaine hydrochloride with the same instructions. Both groups also received 500 mg acetaminophen tablets and preservative-free 1% chloramphenicol antibiotic eye drops to be taken in addition to their assigned treatment. The study concluded that topical tetracaine did not result in a delay of corneal healing (*P *= .761). However, on VAS ratings, there was no statistically significant difference in pain scores between the two groups. The average difference in reported pain over 48 hours was 0.53 on a 100 mm VAS (*P* = .149). Despite this, the patients in the treatment group reported that on a scale of 0-10 of effectiveness, tetracaine was rated 7.7. The patients in the control group reported the saline as 3.8 on a scale of 0-10 effectiveness. Thus, patients felt that the tetracaine was more effective (*P *< .0005).

### CYCLOPLEGICS

Cycloplegics are another class of intervention that can be administered for corneal abrasions; however, the existing literature is limited. Table 3 describes the most current knowledge about the efficacy of cycloplegics on corneal abrasion recovery.

**Table 3:** Study Characteristics and Outcomes of Cycloplegics Trials

Paper Title	Author	Design	Number of Patients	Number on Intervention	Number on Control	Intervention	Outcomes	Followup	Results
Is Homatropine 5% effective in reducing pain associated with corneal abrasion when compared with placebo? A randomized controlled trial	Meek et al. [7]	Randomized controlled trial	40	20	20	Homatropine 5% eye drops applied every 6 hours until 18 hours	Pain using 10 cm VAS pain scale	Evaluation before treatment; evaluation 6, 12, 18, and 24 h after initial treatment; evaluation in person, through mail, at end of treatment	No significant difference in reduction of pain between control and treatment group

A triple-blind, randomized controlled trial conducted by Meek et al. in 2010 investigated the effect of 5% homatropine eye drops compared with 0.5% hypromellose placebo eye drops on pain ratings [7]. Pain was assessed using VAS pain ratings at 6 hour intervals for a 24 hour period during which the study drug was introduced at 0, 6, 12, and 18 hours. A significant reduction in pain was defined as a greater than 20mm decrease from the time of enrollment to each time point. Forty patients were recruited as a convenience sample from an urban district hospital and were randomly assigned to one of the two treatment groups - 20 patients received homatropine, and 20 patients received placebo treatment. Eligible patients required a sustained mechanical corneal abrasion within the previous 12 hours. Exclusion criteria included the following: ocular intervention or application of topical eye medication within the prior 48 hours; corneal ulceration secondary to non-mechanical trauma, such as chemical exposure or viral infection, or associated with contact lens use; pregnant or breastfeeding patients; patients with Parkinson’s Disease, glaucoma, keratoconus, or astigmatism. Baseline characteristics between the two groups were not significantly different from each other; these variables included age, sex, attendance, mechanism of injury, and time of presentation, among others. Although 50% (95% CI, -27.2-72.8) of patients in the homatropine group and 60% (95% CI, -36.1-80.9) of patients in the placebo group reported a greater than 20mm VAS decrease in pain at 12 hours, pain score reductions between the two groups were not significantly different from each other at any time point (*P *> .05). Meek et al. concluded that cycloplegics should not be considered for routine use but may be considered if ciliary muscle spasm or intraocular inflammation is present.

## Conclusions

While topical ophthalmologic solutions are commonly given to patients presenting to the emergency department for corneal abrasions, there has been limited progress in establishing specific guidelines to stratify the best treatment option. Patients with corneal abrasions are typically treated with a minimum of topical antibiotics to reduce the risk of infection, but the protocol for optimal pharmacological pain management remains a topic of debate. Three main classes of topical medication have been explored: NSAIDs, anesthetics, and cycloplegics. Trials involving the use of NSAIDs for pain management in corneal abrasion have shown a statistically significant decrease in subjective pain experienced by the patient. However, the efficacy of topical anesthetics on pain control in corneal abrasions is unclear and requires more studies. The current literature  involving topical anesthetics suffers from small sample sizes. Because of the limitations of the available studies, it is difficult to recommend or discourage the use of topical anesthetics for pain control. The sole published study on cycloplegics showed no significant pain reduction; therefore, further studies are necessitated before this intervention can be encouraged for use in corneal abrasions. Thus, based on this review of the current literature, NSAIDs and proparacaine, an anesthetic agent, have been shown to be the most effective topical interventions for subjective pain reduction in corneal abrasions. Additionally, NSAIDs have been shown to have no effect on the rate of corneal epithelial growth after an abrasion. Because these studies investigating the efficacy of topical analgesia for corneal abrasions are limited by small sample sizes, the current available data, especially regarding topical anesthetics and cycloplegics, is too limited to draw recommendations for management. As a result, large multi-center randomized controlled trials should be performed in order to properly demonstrate effective pain control.
